# Construction of an infectious clone of tomato leaf curl New Delhi virus and development of a duplex assay through recombinase polymerase amplification-lateral flow dipstick to detect the virus and tomato yellow leaf curl virus simultaneously

**DOI:** 10.3389/fmicb.2026.1940758

**Published:** 2026-09-30

**Authors:** Ning Qiao, Yuxing Chen, Yao Meng, Xiao Yang, Xin Wang, Lihao Zhou, Huijie Dai, Xiaoan Sun, Xiumei Ni

**Affiliations:** 1 Shandong Provincial University Laboratory of Green Diseases and Pests Management in Protected Vegetables, Weifang University of Science and Technology, Shouguang, Shandong, China; 2 School of Life Sciences, Yunnan Normal University, Kunming, Yunnan, China; 3 Shandong Vocational Animal Science and Veterinary College, Weifang, Shandong, China

**Keywords:** infectious clone, lateral flow dipstick, recombinase polymerase amplification, tomato leaf curl New Delhi virus, tomato yellow leaf curl virus, viral co-infection

## Abstract

The co-infection of tomato yellow leaf curl virus (TYLCV) and tomato leaf curl New Delhi virus (ToLCNDV) in the same host complicates disease diagnosis and management, not to mention that each virus alone can cause devastating losses to its respective host crops across many tomato- and cucurbit-growing areas. However, diagnosis of mixed viral infections in the same host plants can be tedious, costly and challenging. This study aims to establish a rapid, sensitive, and specific duplex recombinase polymerase amplification-lateral flow dipstick (RPA-LFD) assay to simultaneously detect TYLCV and/or ToLCNDV in co-infected samples. We constructed an infectious clone from a ToLCNDV strain of the Asian clade isolated from a diseased cucumber plant in China, and confirmed that it could co-infect *Nicotiana benthamiana* together with an infectious clone of the TYLCV-IL strain, providing the defined viral inoculum for method development and validation. An optimal duplex RPA-LFD assay was developed by screening specific RPA primers and probes targeting conserved sequences of both viruses and optimizing reaction parameters, which produced interpretable results directly on a test strip after 20 min at 39 °C. With the purified plasmid DNA, the analytical limits of detection of the duplex RPA-LFD for TYLCV and ToLCNDV were 10^2^ and 10^1^ copies/μL, respectively. No cross-reactions were detected against three other begomoviruses. Further validation using total DNA from co-inoculated *N. benthamiana* and field-collected samples showed high consistency with conventional PCR, and the assay effectively distinguished single-virus from mixed infections. This duplex RPA-LFD assay provides a reliable diagnostic for detecting and surveying co-infections caused by TYLCV and ToLCNDV.

## 1 Introduction

Members of the genus *Begomovirus* (family *Geminiviridae*) are important viruses responsible for serious diseases of crops in many tropical and subtropical regions (Varma and Malathi, 2003; Rojas et al., 2018). Among them, tomato yellow leaf curl virus (TYLCV) and tomato leaf curl New Delhi virus (ToLCNDV) are two of the most widely and economically destructive viruses (Charoenvilaisiri et al., 2020; Xie et al., 2025). TYLCV primarily infects tomatoes but can extend its invasions to other crops such as cucumber to cause yellowish and curled leaves, stunted growth, and significant yield loss (Gelbart et al., 2020). ToLCNDV has a broader host range including tomatoes and other cucurbit crops (e.g., cucumbers, squashes, and melons) as well as peppers and tobacco. Its infection induces leaf curling, yellowing, mosaic, and stunting, which are typical symptoms of begomovirus-caused diseases (Moriones et al., 2017). Both viruses are transmitted persistently and circulatively by the whitefly (*Bemisia tabaci*), making it difficult to curb their continuous inoculations due to the unpredictable and explosive nature of whitefly populations (Idrees et al., 2024).

As both viruses have overlapping tomato and cucurbit hosts, mixed infections occur frequently in the field. Indeed, TYLCV–ToLCNDV co-infections have been detected at high prevalence in field surveys—up to ~50% of symptomatic tomato samples (Vo et al., 2022)—underscoring the practical need for methods capable of simultaneously detecting both viruses. Therefore, it is essential to develop a rapid, accurate, sensitive, cost-effective, and robust assay for simultaneous detection of both viruses in co-infected hosts (Singhal et al., 2021).

Serological tests such as the enzyme-linked immunosorbent assay (ELISA) and molecular assays like polymerase chain reaction (PCR), real-time PCR, and loop-mediated isothermal amplification (LAMP) are widely used for detection of plant viruses (Roy et al., 2024; Singh et al., 2025; Nguyen et al., 2026). However, ELISA is relatively low in its sensitivity and the preparation of high-quality monoclonal antisera can be complex and difficult. Although conventional PCR and its variants are sensitive and specific, they require precise thermal cycling instruments and are time-consuming. These constraints have limited their use in resource-inadequate laboratories or field settings. Instead, LAMP is an established isothermal approach that amplifies nucleic acids in a single step without thermal cycling, however, its primer design can be intricate and its development of a multiplex detection system remains challenging (Tighe et al., 2015; Yang et al., 2024; Xie et al., 2025). Recombinase polymerase amplification (RPA) utilizes the recombinase, single-stranded DNA-binding proteins, and strand-displacing DNA polymerase to amplify target sequences in 20 min at a constant temperature between 37 and 42 °C (Munawar, 2022; Wu et al., 2024). Coupled with the RPA procedure, a lateral flow dipstick (LFD) can also be developed to visualize the amplicons labeled with specific tags (e.g., fluorescein amidite [FAM] and biotin) through antigen-antibody reactions. The RPA-LFD “amplify-and-read” assay is fast, convenient, and efficient to be used for detecting multiple pathogens without sophisticated equipment (Dai et al., 2019; Liao et al., 2024).

Although a singleplex RPA assay for either TYLCV or ToLCNDV and a duplex RPA detection of both viruses based on gel electrophoresis have been reported (Zhou et al., 2022; Roy et al., 2025; Yuan et al., 2026), a duplex RPA-LFD assay for the simultaneous visualization of both viruses has not been established. Infectious clones can provide defined and specific viral inocula to minimize interferences from genetic variations, inhibitors, and fluctuating viral titers during method development and validation (Pasin et al., 2019; Saad et al., 2021; Shakir et al., 2023; Moller and Maruthi, 2025). Although infectious clones of many begomoviruses including mungbean yellow mosaic India virus (MYMIV) (Kumari et al., 2024), cotton leaf curl virus (CLCuV) (Briddon et al., 2000), and TYLCV (Zhang et al., 2009) have been successfully constructed, and an infectious clone of ToLCNDV from melon in Jiangsu has been reported (Mei et al., 2023), infectious clones of the Asian clade isolated from cucurbits in northern China remain unavailable, and the combined use of infectious clones with RPA-LFD for method validation has not been explored. To address these gaps, this study aimed to construct an infectious clone of a ToLCNDV strain from the Asian clade isolated from cucurbits, validate its co-infectivity with a TYLCV infectious clone in *Nicotiana benthamiana*, and develop a duplex RPA-LFD assay for the simultaneous detection of both viruses.

## 2 Materials and methods

### 2.1 Virus sources and plant materials

The TYLCV infectious clone (pCB301-TYLCV, TY301) of the TYLCV-IL strain constructed by Zhejiang University (Zhang et al., 2009) was kindly provided. A ToLCNDV strain of the Asian clade isolated from field cucumber samples collected in Shandong Province (Qiao et al., 2026) was used to construct its infectious clone. Other begomoviruses, such as tomato leaf curl virus (TLCV), cotton leaf curl multan virus (CLCuMuV) and squash leaf curl China virus (SLCCNV) were provided by Dr. L. Zang's laboratory at Shandong Agricultural University. Seeds of *N. benthamiana* were maintained in our laboratory. Seeds of tomato (*Solanum lycopersicum*), cucumber (*Cucumis sativus*), pumpkin (*Cucurbita moschata*), zucchini (*Cucurbita pepo*), watermelon (*Citrullus lanatus*), smooth luffa (*Luffa cylindrica*), and melon (*Cucumis melo*) were purchased from a local seed company. All seeds were surface-sterilized and sown in pots with sterilized potting mix. The plants were cultivated in an artificial climate chamber at 25 ± 2 °C under a 16 h light/8 h dark photoperiod.

### 2.2 Construction of ToLCNDV infectious clone

Based on previously obtained full-length sequences of DNA-A and DNA-B (GenBank accession numbers: PV083591.1 and PV083592.1) of the above-mentioned ToLCNDV isolate, the tandem repeat fragments containing approximately 1.4-mer of the DNA-A and 1.7-mer of the DNA-B genome were amplified by PCR. The PCR reaction mixture contained: 10.0 μL of 2 × TransStart^®^ FastPfu PCR SuperMix (TransGen Biotech Co., Ltd., Beijing, China), 0.5 μL of forward and reverse primers, approximately 50 ng of DNA-A or DNA-B template, and ddH_2_O to a final volume of 20.0 μL. The PCR amplification procedure was set as: 95 °C for 3 min followed by 35 cycles of 95 °C for 30 s, 58 °C for 30 s, 72 °C for 2–3 min (adjusted according to fragment size, calculated as 1 kb/min), and 72 °C for 5 min for the final extension. Amplicons were analyzed by agarose gel electrophoresis and purified using the EasyPure^®^ Quick Gel Extraction Kit (TransGen). Utilizing the ClonExpress Ultra One-Step Cloning Kit (Vazyme Biotech Co., Ltd., Nanjing, China) based on homologous recombination, purified tandem repeat fragments were individually cloned into the plant binary vector pCB301, constructing infectious clones containing DNA-A (named ND-A301) and DNA-B (named ND-B301). Recombinant plasmids were transformed into *E. coli* DH5α competent cells. Positive clones were screened by the colony PCR and verified by sequencing and then stored at −20 °C. Primers used for constructing the ToLCNDV infectious clone and subsequent research were synthesized by Tsingke Biotechnology Co., Ltd. (Beijing, China) (sequences listed in Table 1). Three recombinant plasmids (TY301, ND-A301, and ND-B301) were extracted using the EasyPure^®^ Plasmid MiniPrep Kit (TransGen). The plasmid concentration was measured using Nanodrop to calculate the number of copies using the formula: Copies/μL = [plasmid concentration (ng/μL) × 6.02 × 10^23^] / [plasmid length (bp) × 1 × 10^9^ × 660].

**Table 1:** Primers designed for RPA and PCR assays for ToLCNDV and TYLCV.

Primer^*^	Length (bp)	Sequence (5^′^-3^′^)	Application
ToND(A)301-1-F	41	CATTTCATTTGGAGAGGCCTGGCCCCCATGAATTCTTTAAA	Construction of an infectious clone of ToLCNDV DNA-A
ToND(A)301-1-R	34	GGATCCCACATGTTTGTGGATCCAAACTTGGTGA	
ToND(A)301-2-F	33	TCCACAAACATGTGGGATCCATTATTACACGAA	
ToND(A)301-2-R	42	GGGGATCCTCTAGAGTCGACCCACATGTTTGTGGATCCAAAC	
ToND(B)301-1-F	50	CATTTCATTTGGAGAGGCCTGCAGAAGAAACATTTTATTGATAAATAAAT	Construction of an infectious clone of ToLCNDV DNA-B
ToND(B)301-1-R	34	GAAACGCCATTTGGCTAGGATAGGAAATGTGTGG	
ToND(B)301-2-F	32	TCCTAGCCAAATGGCGTTTCCCTCTCCTTATT	
ToND(B)301-2-R	40	GGGGATCCTCTAGAGTCGACCGCCATTTGGCTAGGATAGG	
ND-PCR-F	22	GAATCTAGGCACGATGTCTCTC	PCR for ToLCNDV detection (Amplicon size is 475 bp)
ND-PCR-R	22	CGTGAGTACAGGCCATATACAA	
TY-PCR-F	23	ACGCATGCCTCTAATCCAGTGTA	PCR for TYLCV detection (Amplicon size is 543 bp)
TY-PCR-R	24	CCAATAAGGCGTAAGCGTGTAGAC	
ND-RPA-F	30	TCTCATAGCATCGTCTTCATTTCCCGTGTT	RPA for ToLCNDV detection
ND-RPA-R/ND-RPA-R (5′-Bio)	30	GGTCTCCGCATCTCAATGTTCTTATCCAAT	
ND-probe-FAM	18	GGGCATAGGCATCGTTTG	
TY-RPA-F	28	GAAAACTCCAAAATCAATGAAGACTCCG	RPA for TYLCV detection
TY-RPA-R/TY-RPA-R (5′-Dig)	28	AAGGCAAATACCAATGTAAGAAGCAACG	
TY-probe-FAM	17	GCTCGGATGGAAATGTG	

^*^All primers were synthesized by Tsingke Biotechnology Co., Ltd., Beijing, China.

### 2.3 Agroinfiltration inoculation and virus infectivity assay

The plasmids TY301, ND-A301, and ND-B301 were separately transformed into *Agrobacterium tumefaciens* strain GV3101. *Agrobacterium* cultures containing ND-A301 and ND-B301 were mixed in equal volumes and resuspended in infiltration buffer (10 mM MES, 10 mM MgCl_2_, 150 μM acetosyringone, pH 5.6) to an OD_600_ of 1.0. Four- to five-leaf-stage seedlings of *N. benthamiana*, tomato, and various cucurbit crops were infiltrated through a needleless syringe. After inoculation, the plants were maintained in a climate chamber, and symptoms were observed and recorded regularly. Total plant DNA was extracted using the FastPure^®^ Plant DNA Isolation Mini Kit (Vazyme) and stored at −20 °C until further use. In addition, *N. benthamiana* plants were agroinfiltrated with *Agrobacterium* cultures containing the TYLCV infectious clone either alone or in combination with the mixed ToLCNDV cultures described above, establishing treatments including a healthy control, single-virus infection, and co-infection. Systemic leaves were collected from each treatment group at 10 days post-inoculation, and total plant DNA was extracted and stored for subsequent analysis.

PCR detection was performed using specific primer pairs (ToLCNDV: ND-PCR-F/R; TYLCV: TY-PCR-F/R) (Table 1). The reaction mixture contained 10.0 μL of 2 × EasyTaq^®^ PCR SuperMix (Vazyme), 0.5 μL of each primers, 1.0 μL of template DNA, and ddH_2_O to 20.0 μL. The PCR procedure was set as 95 °C for 3 min, 30 cycles of 95 °C for 15 s, 56 °C for 15 s, 72 °C for 30 s, and 72 °C for 5 min as the final extension. The amplicons were then subjected to agarose gel electrophoresis for further analysis.

### 2.4 Design and synthesis of RPA primers and LFD probes

Sequence alignment was performed using the complete nucleotide sequences of Chinese TYLCV isolates and ToLCNDV DNA-A isolates from GenBank described in details in Supplementary Table S1 for their host origin, geographic location, and accession numbers. The software used for this analysis was MEGA11 (Tamura et al., 2021). Conserved regions in the sequences of both viruses were selected to design duplex RPA-specific primers and probes (Table 1). Probes were labeled with FAM, biotin, or digoxigenin (DIG). All primers and probes were synthesized, diluted to 10 μM, and stored in a −20 °C freezer.

### 2.5 Singleplex basic RPA for ToLCNDV and TYLCV

The validation of primer efficacy was performed by the singleplex basic RPA reactions following the instructions provided in the TwistAmp^®^ Basic Kit (TwistDx Ltd., Cambridge, UK). The master mix contained 29.5 μL of rehydration buffer, 2.0 μL each of forward and reverse primers, 2.0 μL of TY301 or ND-A301 plasmid template (approximately 10^6^ copies/μL), and 12.0 μL of ddH_2_O in a centrifuge tube containing the RPA freeze-dried enzyme pellets. All ingredients in the tube were thoroughly mixed to ensure complete dissolution before 2.5 μL of 280 mM magnesium acetate (MgOAc) was added to initiate the reaction for 25 min at 39 °C in water bath or on a heating block. The incubation was terminated by placing the reaction tube on ice. The amplicons were purified using the EasyPure^®^ PCR Purification Kit (TransGen) and analyzed through a 2% agarose gel electrophoresis. Each reaction was independently repeated three times.

### 2.6 Establishment of duplex basic RPA and optimization of primer ratio

In order to optimize the duplex RPA reaction and determine the ratio of primers of TYLCV and ToLCNDV, the two pre-mixed virus-specific primer pairs (TY-RPA-F/R and ND-RPA-F/R) were tested at volume ratios of 1:3, 1:2, 1:1, 2:1, and 3:1. The master mix included 29.5 μL of primer-free dehydration buffer, 2.0 μL each of TY301 and ND-A301 plasmid templates, 4.0 μL of mixed primers at various ratios, 10.0 μL of ddH_2_O, and 2.5 μL of MgOAc. Reactions were carried out as described above. Each primer ratio was tested in triplicate.

### 2.7 Optimization of duplex RPA reaction temperature and time

To ascertain the optimal reaction temperature and time, reactions were conducted at 35, 37, 39, 41, and 43 °C for 25 min at the optimal primer ratio, and at 39 °C for 10, 15, 20, 25, and 30 min respectively. All ingredient preparation, reaction setup and amplicon analysis were performed as described above. Each temperature and time condition was tested in triplicate.

### 2.8 Establishment of duplex RPA-LFD and optimization of probe/primer combinations

The RPA-LFD protocol was established through applying the optimized duplex RPA system incorporated with labeled probes. The volume ratios of the TYLCV and ToLCNDV probes (TY-probe-FAM:ND-probe-FAM) were set at 1:2, 1:1, and 2:1. A 50.0 μL total volume of reaction ingredients included 29.5 μL of primer-free rehydration buffer, 2.0 μL each of TY301 and ND-A301 plasmid templates, 1.4 μL each of TY-RPA-F and TY-RPA-R-Dig, 0.7 μL each of ND-RPA-F and ND-RPA-R-Bio, 1.0 μL of mixed probes at different ratios, and 8.8 μL ddH_2_O. Finally, 2.5 μL of MgOAc was added to the mixed and touch-centrifuged reagents to initiate the reaction. After an incubation for 20 min at 39 °C, 15.0 μL of amplicons was then combined with 90.0 μL of assay buffer. The tip of the sample pad of the dual-target lateral flow dipstick (Jieshi East Biotech, Xiamen, China; Figure 1) was dipped vertically into the mixture; the result was read after 5 min. The remaining amplicons were subject to agarose gel electrophoresis as a control. Each probe ratio was tested in triplicate.

**Figure 1:**
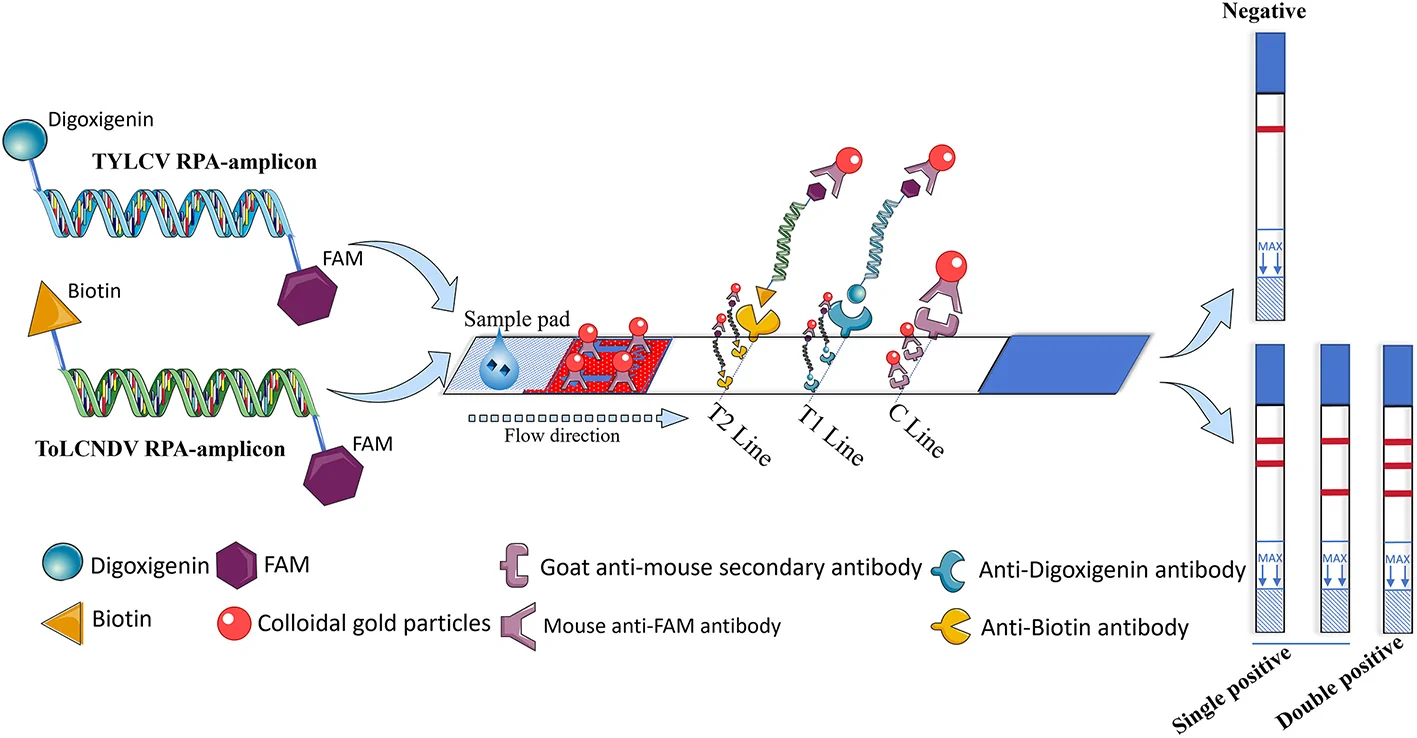
Visualization principle and result interpretation of the dual-target lateral flow dipstick (LFD). The schematic illustrates the color development mechanism, antibody coating pattern, and representative positive and negative results on the dual-target LFD strip.

### 2.9 Sensitivity analysis of the duplex RPA-LFD

The TY301 and ND-A301 plasmids were mixed at a 1:1 molar ratio and subjected to 10-fold serial dilutions (10^6^-10^0^ copies/μL). Each dilution was tested in three technical replicates under optimized reaction conditions for duplex RPA-LFD detection, and the limit of detection (LOD) was defined as the lowest concentration at which all three replicates were consistently and clearly positive. Concurrently, conventional PCR testing was performed (using the primers and conditions previously described) on the same dilution series as comparisons.

### 2.10 Specificity test of the duplex RPA-LFD

The specificity of the duplex RPA-LFD was evaluated using the following templates: TY301 plasmid (TYLCV), ND-A301 plasmid (ToLCNDV), mixed TY301+ND-A301 plasmids (double positive), genomic DNA from other begomoviruses including TLCV, CLCuMuV, and SLCCNV, total DNA from healthy *N. benthamiana* plants. All validation procedures were performed in accordance with the aforementioned methods. Each template was tested in triplicate.

### 2.11 Validation and application of the duplex RPA-LFD

The optimized duplex RPA-LFD protocol was used to detect target DNAs from 40 *N. benthamiana* plants (10 per treatment across four treatments): the plants were infiltrated with the empty vector (mock control), the TYLCV infectious clone, the ToLCNDV infectious clone, or a mixture of both infectious clones. To further streamline the workflow, total DNA was also rapidly extracted using QuickExtract^TM^ Plant DNA Extraction Solution (LGC Biosearch Technologies, Alexandria, VA, USA). Briefly, leaf discs (4–6 mm in diameter) were collected from each sample and placed in 100 μL of QuickExtract solution. The mixture was heated at 65 °C for 6 min, followed by 98 °C for 2 min according to the manufacturer's instructions. A 2 μL aliquot of the extract was used as the template for the optimized duplex RPA-LFD assay (see Figure 2 for workflow). Conventional PCR was performed in parallel using the same QuickExtract-prepared DNA as the reference method. Additionally, a total of 57 field-collected samples (21 tomato and 36 cucurbit plants) with symptoms suggestive of begomovirus infection were tested using both the RPA-LFD and conventional PCR protocols described above. The agreement rate (%) was calculated as: (Number of samples for which the two methods gave a concordant infection status—i.e., both positive for at least one virus or both negative for both viruses—divided by the total number of samples) × 100%. The sensitivity, specificity, positive predictive value, and negative predictive value were calculated with the conventional PCR as the reference method, and agreement between the two assays was quantified using Cohen's kappa (Cohen, 1960), interpreted according to Landis and Koch (1977).

**Figure 2:**
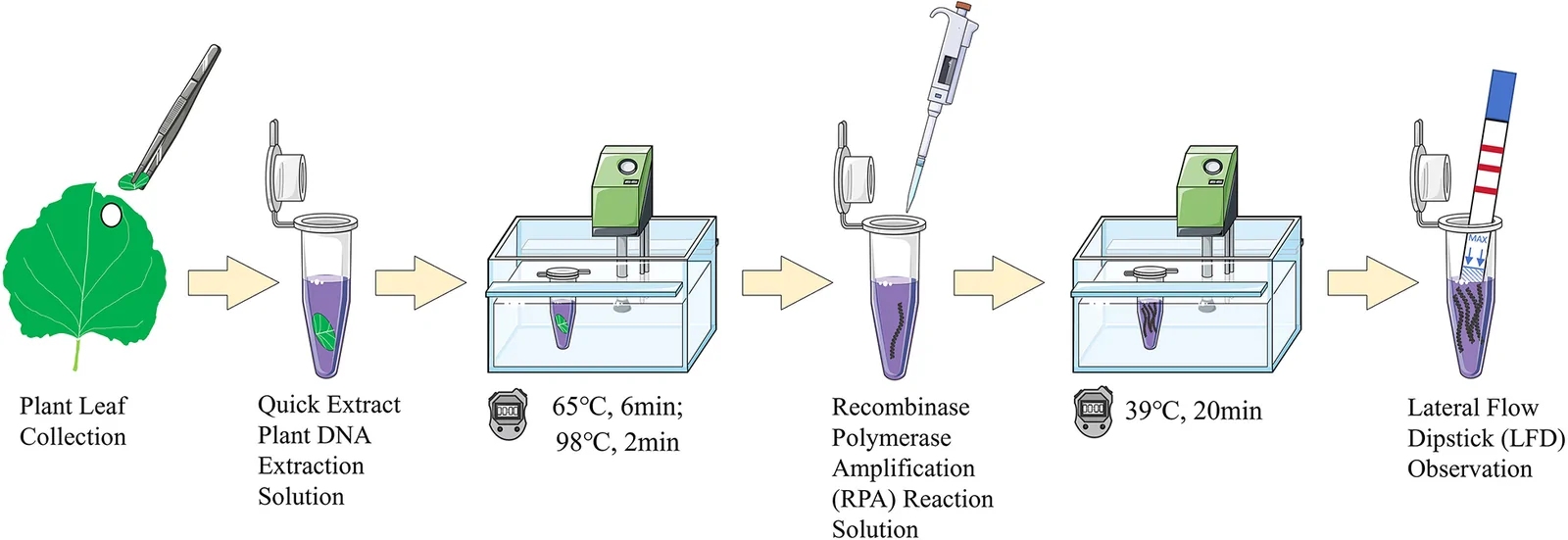
Workflow of rapid DNA extraction coupled with duplex RPA-LFD detection.

To independently confirm the TYLCV–ToLCNDV co-infections, the conventional PCR amplicons (TYLCV and ToLCNDV targets, amplified with TY-PCR-F/R and ND-PCR-F/R; Table 1) from the four double-positive field tomato samples were gel-purified and ligated into the pEASY-T1 Cloning Vector (TransGen). Following the same cloning and screening procedure described above, the positive clones were subjected to bidirectional sequencing with M13 universal primers. The resulting sequences were analyzed by BLAST against the corresponding TYLCV and ToLCNDV reference sequences to confirm their identities.

## 3 Results

### 3.1 DNA-A and DNA-B infectious clones of a cucurbit-isolated ToLCNDV strain and their biological characteristics

With a homologous recombination strategy, plasmid ND-A301 and ND-B301 containing tandem repeats of ~1.4-mer of ToLCNDV DNA-A and ~1.7-mer of DNA-B were successfully constructed (Figure 3A). 8–10 days after infiltration of a mixture of both clones into *N. benthamiana* leaves, systemic viral symptoms of curling, wrinkling, mild yellowing, and stunting appeared on newly emerged leaves (Figure 3B). After 10–14 days of inoculation on cucurbit crops, ToLCNDV successfully infected cucumber, pumpkin, zucchini, watermelon, smooth luffa, and melon, caused similar symptoms of leaf curling, distortion, and mosaic (Figures 3C–H). All mock-inoculated (empty vector) control plants (Supplementary Figure S1) and healthy control plants exhibited no symptoms at all. Under the experimental conditions, all 21 inoculated tomato plants remained asymptomatic throughout the entire experiment (Figure 3I).

**Figure 3:**
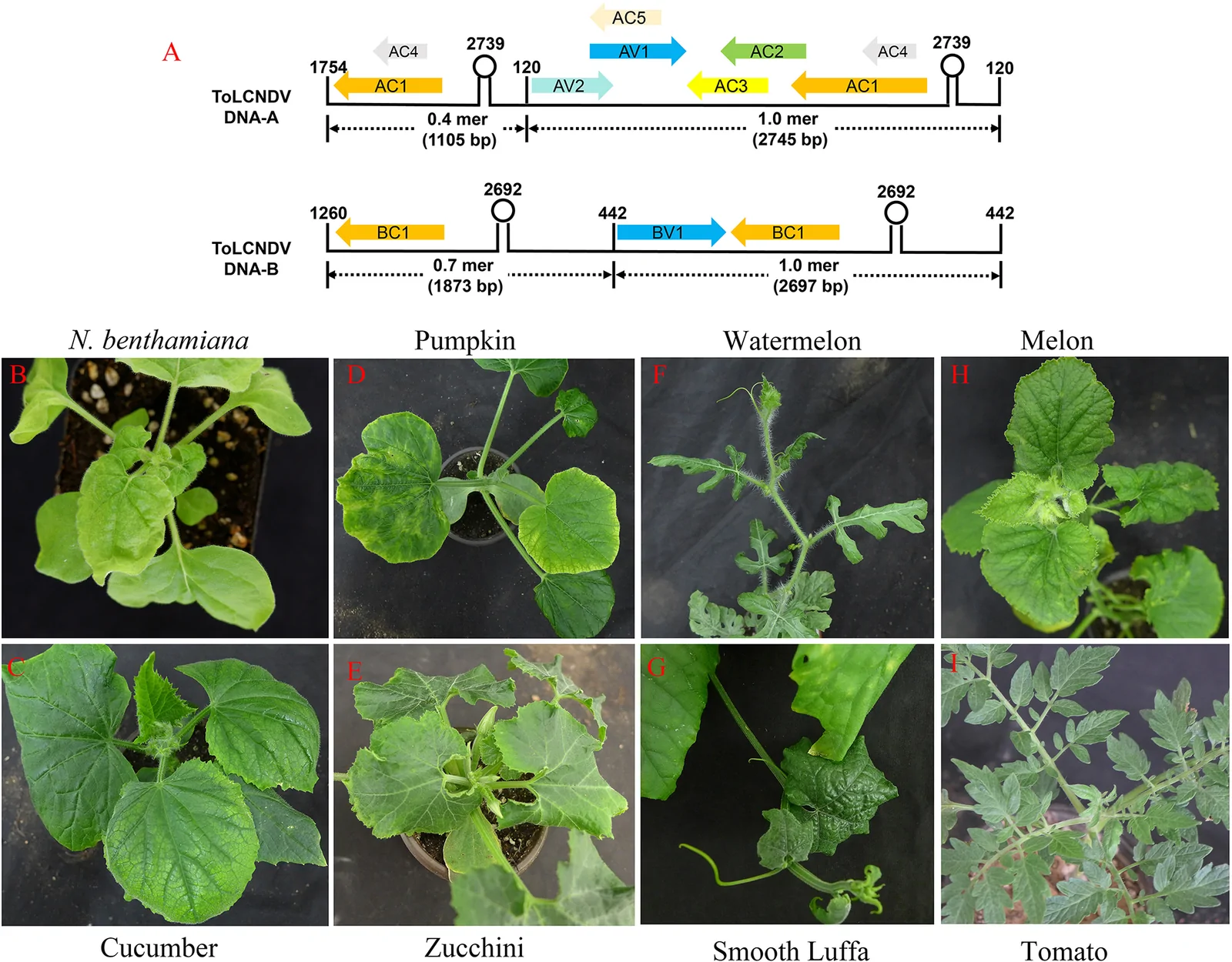
Construction of the ToLCNDV infectious clone and expression of symptoms after inoculation. **(A)** Schematic diagram illustrating the construction strategy for the ToLCNDV infectious clones (ND-A301 and ND-B301). **(B)**–**(I)** Typical symptoms induced by ToLCNDV inoculation on *N. benthamiana* (**B**), cucumber (**C**), pumpkin (**D**), zucchini (**E**), watermelon (**F**), smooth luffa (**G**), and melon (**H**), including leaf curling, crinkling, yellowing, and mosaic. No obvious symptoms were observed on tomato (**I**).

Successful infection by the infectious clones was confirmed by conventional PCR, which amplified specific ToLCNDV bands from all symptomatic *N. benthamiana* and cucurbit plants, whereas no viral DNA was detected in any mock controls. Notably, 3 out of 21 inoculated tomato plants were PCR-positive despite the absence of visible symptoms, giving a low infection rate of 14.3%. The infection rates of ToLCNDV on *N. benthamiana*, cucumber, pumpkin, zucchini, watermelon, smooth luffa, and melon were 100% (15/15), 85.7% (12/14), 93.3% (14/15), 83.3% (10/12), 61.5% (8/13), 83.3% (10/12), and 86.7% (13/15), respectively (Table 2). These results indicate that the constructed ToLCNDV clones are biologically infectious to cucurbit crops and *N. benthamiana*, and could establish a low-rate, asymptomatic infection in the tomato cultivars tested.

**Table 2:** The infection rate of ToLCNDV infectious clone on different plant species.

Species	No. of inoculated plants	No. of diseased plants	No. of positive plants via PCR	Infection rate (%)
*N. benthamiana*	15	15	15	***100***
Cucumber (*Cucumis sativus*)	14	12	12	***85.7***
Pumpkin (*Cucurbita moschata*)	15	14	14	***93.3***
Zucchini (*Cucurbita pepo*)	12	10	10	***83.3***
Watermelon (*Citrullus lanatus*)	13	8	8	***61.5***
Smooth Luffa (*Luffa cylindrica*)	12	10	10	***83.3***
Melon (*Cucumis melo*)	15	13	13	***86.7***
Tomato (*Solanum lycopersicum*)	21	0	3	***14.3***

### 3.2 Establishment and optimization of the RPA reaction system

The singleplex RPA protocol using the designed specific primers produced clearly separated bands of the expected sizes (284 bp for ToLCNDV and 155 bp for TYLCV), with no cross-reactions between the two primer sets nor background amplifications in uninfected plants (Figure 4A), indicating they were suitable for the duplex RPA detection. In duplex RPA reactions, the 2:1 ratio of TYLCV to ToLCNDV primers produced the most balanced signals for the two target bands and the strongest overall signals (Figure 4B). The primer ratio, amplification temperatures and duration were then optimized. Based on band brightness, clarity, and stability, the optimal conditions for the RPA were at 39 °C (Figure 4C) for 20 min (Figure 4D), although efficient amplifications occurred at the temperature of 35–43 °C and the minimum detectable amplification time was 10 min.

**Figure 4:**
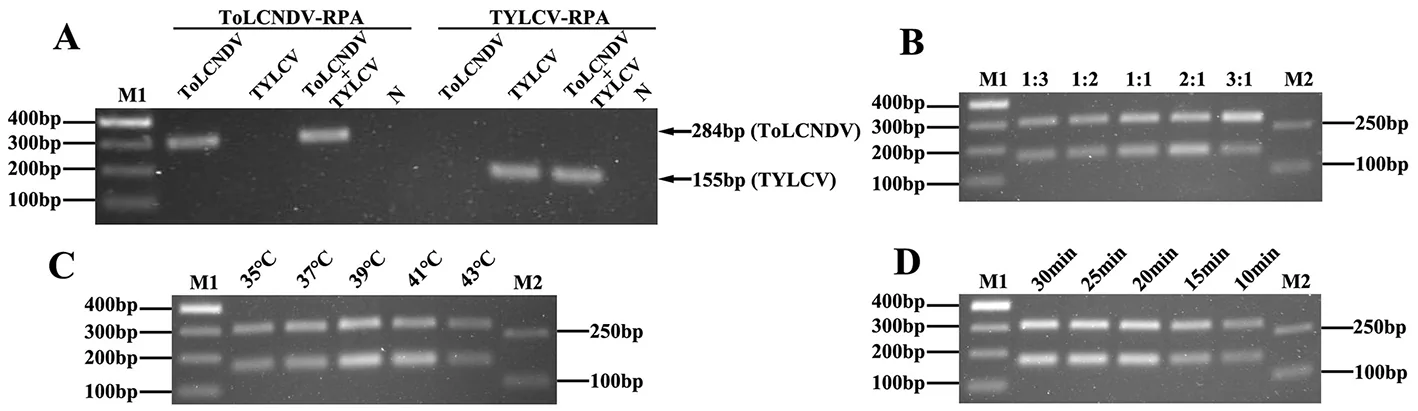
Optimization of duplex RPA reaction conditions. **(A)** Agarose gel electrophoresis of singleplex RPA for ToLCNDV and TYLCV. M1: DNA ladder. N: Negative control (water). **(B)** Duplex RPA gel electrophoresis under different ratios of TYLCV to ToLCNDV primers. **(C)** Duplex RPA gel electrophoresis of amplicons under different amplification temperatures. **(D)** Duplex RPA gel electrophoresis of amplicons under different amplification duration. M1 and M2: DNA ladder.

### 3.3 Establishment of duplex RPA-LFD system for TYLCV and ToLCNDV detection

The RPA-LFD protocol was carried out by incorporating FAM-labeled probes and biotin/DIG-labeled reverse primers into the optimized duplex RPA system. The optimization of the probe concentrations indicated that the 1:1 ratio of probes TY-probe-FAM to ND-probe-FAM rendered the most balanced and clarified signals on the dual-target LFD strip for the T1 line (corresponding to TYLCV) and T2 line (corresponding to ToLCNDV) (Figure 5A), which were further validated by agarose gel electrophoresis of corresponding specific amplification bands (Figure 5B).

**Figure 5:**
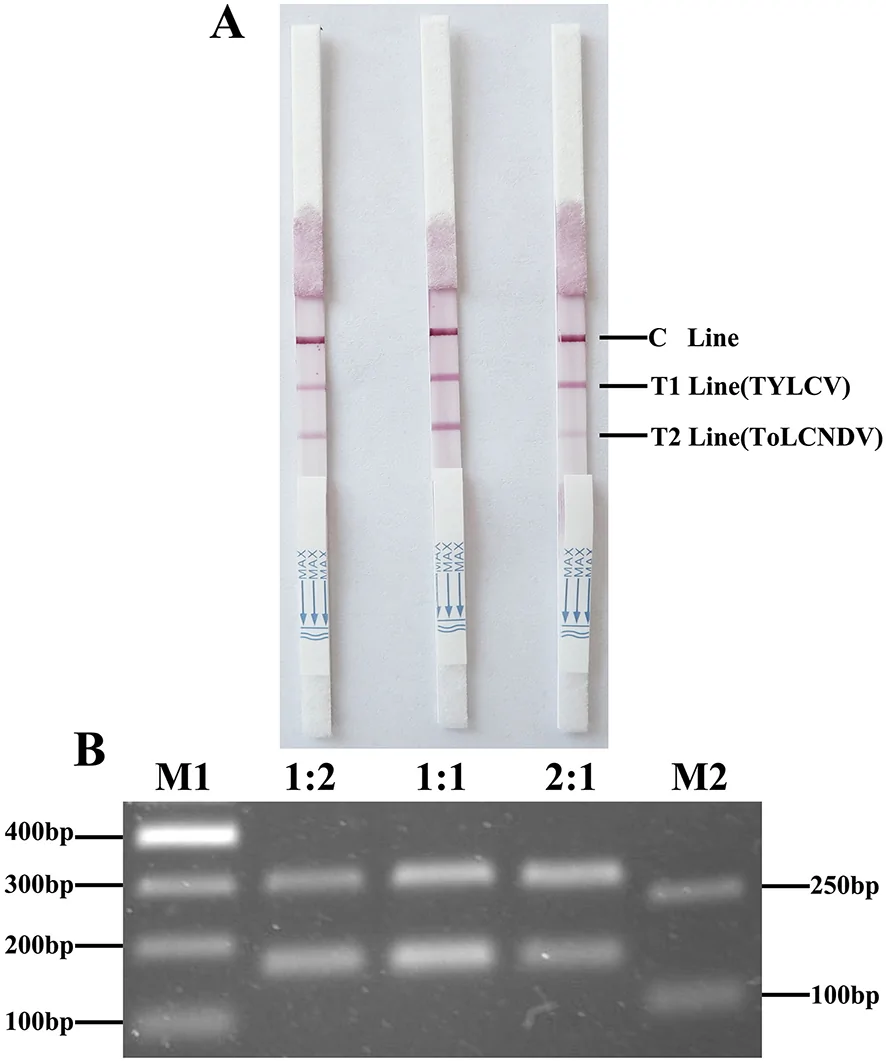
Establishment of duplex RPA-LFD detection system. **(A)** Visualization results of dual-target LFD strips with FAM-labeled probes at different ratios under optimized conditions. C line: Control; T1 line: TYLCV positive control; T2 line: ToLCNDV positive control. **(B)** Agarose gel electrophoresis of RPA amplicons corresponding to samples in panel A. M1 and M2: DNA ladder.

### 3.4 Sensitivity of duplex RPA-LFD detection protocol

The viral detection of mixed plasmids at serial dilutions showed that the analytical LOD (based on purified plasmid DNA) of the duplex RPA-LFD protocol for TYLCV and ToLCNDV detection was 10^2^ copies/μL and 10^1^ copies/μL, respectively (Figures 6A, B). For ToLCNDV, all three technical replicates were positive at concentrations from 10^6^ down to 10^2^ copies/μL (3/3); at 10^1^ copies/μL, 3/3 replicates were positive; at 10^0^ copies/μL, none was positive (0/3). For TYLCV, all three technical replicates were positive at concentrations from 10^6^ down to 10^3^ copies/μL (3/3); at 10^2^ copies/μL, 3/3 replicates were positive; at 10^1^ copies/μL, 0/3 were positive. In comparison, the LOD of conventional PCR was 10^2^ copies/μL for both viruses (Figures 6C, D). The stability of the band signals was compromised once the template concentrations were below the LOD. This outcome suggested that the established duplex RPA-LFD protocol was as sensitive as or slightly more sensitive than conventional PCR in detection of both viruses and their clones.

**Figure 6:**
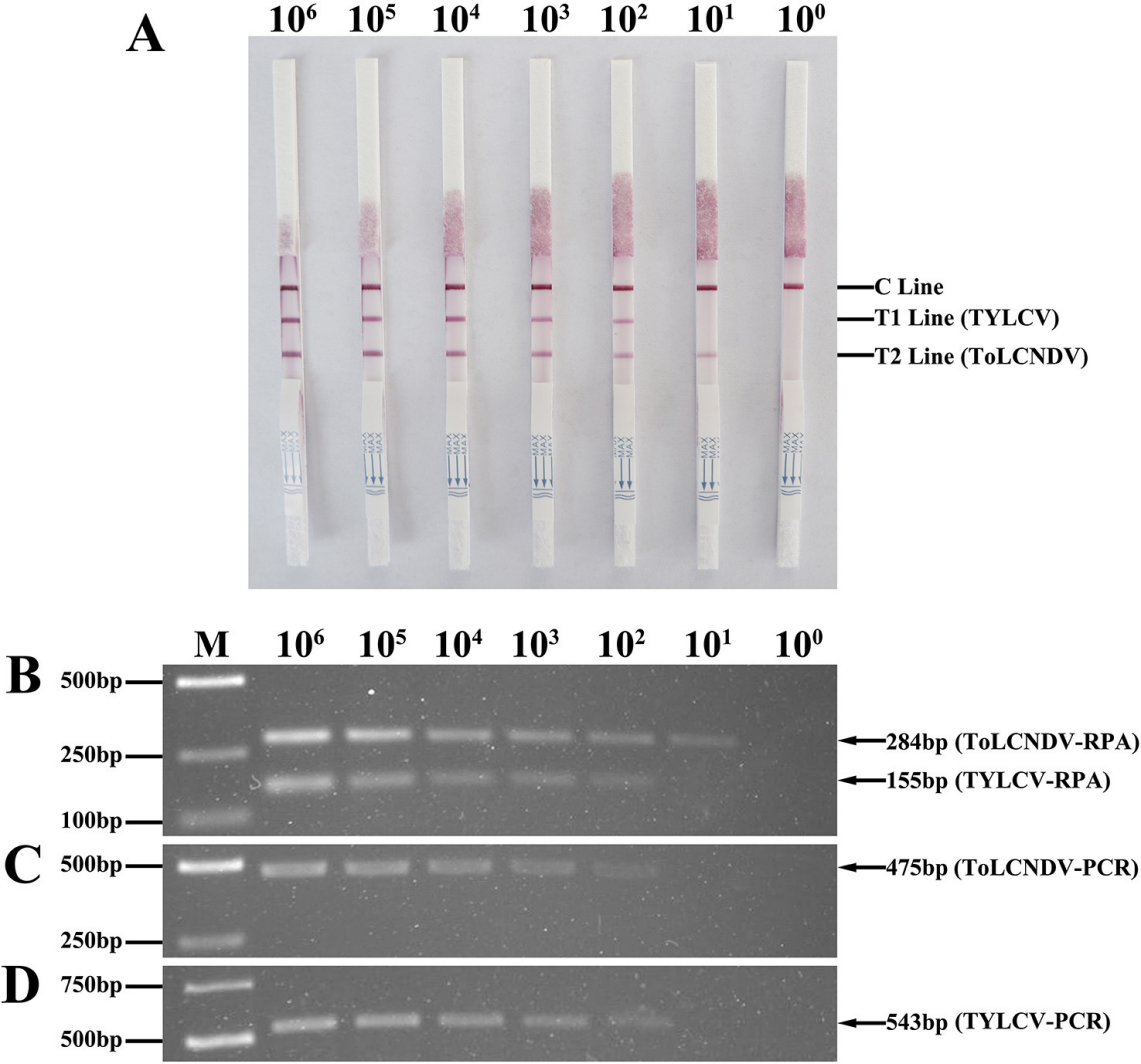
Sensitivity comparison between duplex RPA-LFD and conventional PCR. **(A)** Duplex RPA-LFD detection results for 10-fold serial dilutions of mixed ToLCNDV/TYLCV plasmids. Template concentrations (copies/μL): 10^6^-10^0^. **(B)** Agarose gel electrophoresis of RPA amplicons corresponding to samples in panel A. M: DNA ladder. **(C)** and **(D)** Conventional PCR detection results for the same dilution series of ToLCNDV/TYLCV. M: DNA ladder.

### 3.5 Specificity of duplex RPA-LFD detection protocol

The specificity determination of duplex RPA-LFD detection protocol indicated that the TYLCV template was present and colored in T1 and C lines, while ToLCNDV template showed in T2 and C lines (Figure 7A). When the template was a mixture of both TYLCV and ToLCNDV DNAs, the T1, T2, and C lines were all colored. With the templates derived from other begomoviruses (TLCV, CLCuMuV, SLCCNV) and healthy plant DNA, the C line was colored but not the T1 and T2 lines (Figure 7A). The results from the agarose gel electrophoresis of corresponding PCR amplicons were entirely consistent with those from the LFD (Figure 7B). Therefore, the established duplex RPA-LFD protocol demonstrated high specificity and was accurate in discerning TYLCV from ToLCNDV without cross-reactions with the other begomoviruses tested.

**Figure 7:**
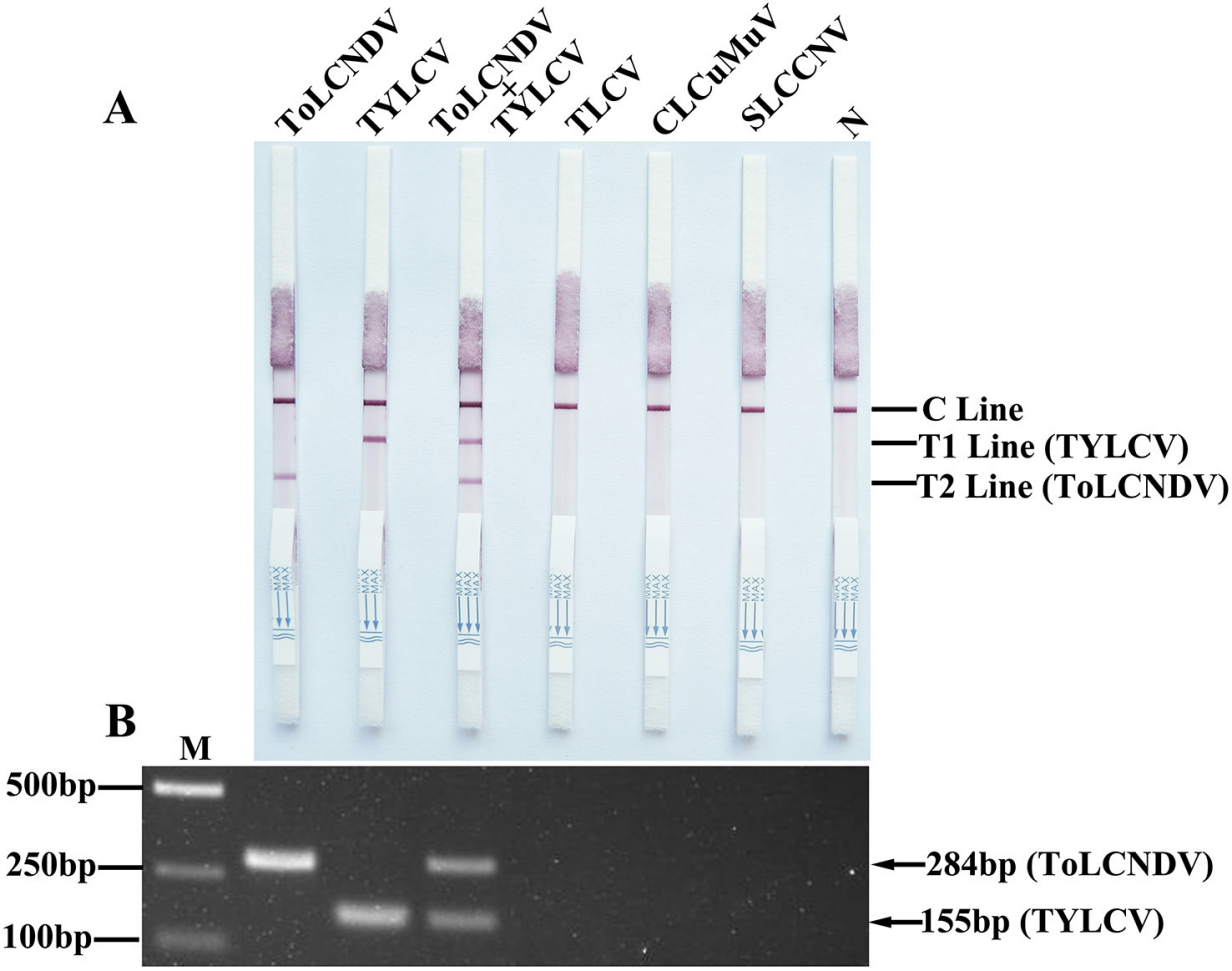
Specificity analysis of the duplex RPA-LFD protocol. **(A)** RPA-LFD detection results for DNA templates of ToLCNDV, TYLCV, and other begomoviruses. N: Healthy *N. benthamiana* DNA. **(B)** Agarose gel electrophoresis of RPA amplicons corresponding to the samples. M: DNA ladder.

### 3.6 Validation of viral detection in inoculated and field-collected samples

The duplex RPA-LFD assay was validated using experimentally co-infected plant material: 40 *N. benthamiana* plants from the mock, TYLCV-alone, ToLCNDV-alone, and co-infected treatments were tested (Figures 8A, B). With the QuickExtract-based DNA preparation, the assay correctly identified all single and mixed infections, with 100% (40/40) concordance with the conventional PCR (Figure 8C), confirming that it reliably distinguishes single from co-infection.

**Figure 8:**
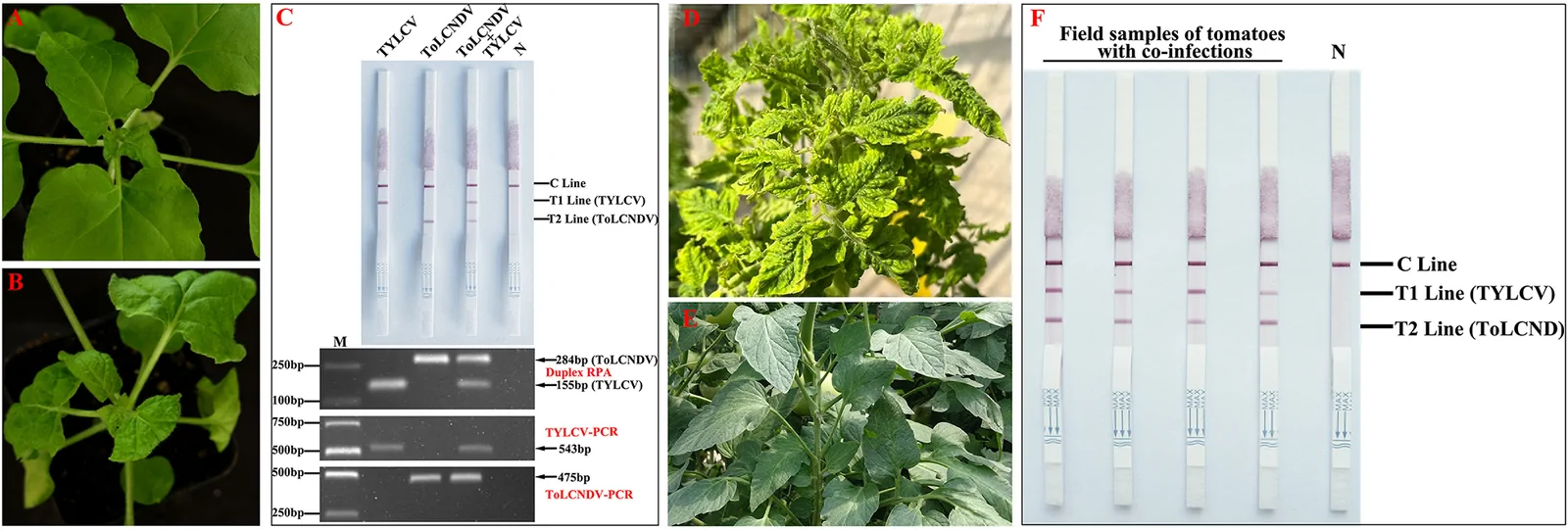
Symptom expression and validation of viral detection in inoculated and field-collected samples. **(A)** TYLCV inoculated *N. benthamiana* showing leaf yellowing and curling; **(B)** *N. benthamiana* co-inoculated with TYLCV and ToLCNDV, showing leaf curling, wrinkling, and yellowing. **(C)** Detection results of duplex RPA-LFD and conventional PCR for samples inoculated with TYLCV and/or ToLCNDV on *N. benthamiana*. N: Healthy *N. benthamiana*; M: DNA ladder. **(D)** Field tomato plant showing symptoms of the TYLCV and ToLCNDV co-infection (yellowing, curling, stunting). **(E)** Healthy tomato plants in the field (negative control). **(F)** Duplex RPA-LFD detection result (showing T1 and T2 double positive) corresponding to the tomato field samples. N: Healthy tomato.

Of 21 field-collected suspect tomato samples, 10 tested positive for TYLCV, 5 positive for ToLCNDV, 4 (~19%) were naturally co-infected with both viruses (Figures 8D, E, F), and 2 tested negative, whereas of 36 suspect cucurbit samples, 2 tested positive for TYLCV, 30 positive for ToLCNDV, and 4 tested negative for both viruses (Table 3). In comparison with the conventional PCR results, the duplex RPA-LFD detection protocol had a 94.7% (54/57) agreement rate. Detailed two-by-two contingency tables and complete diagnostic performance metrics are provided in Table 4. Against conventional PCR as reference, TYLCV detection showed 100% sensitivity and 95.3% specificity, with kappa = 0.91 (almost perfect agreement); ToLCNDV detection showed 100% sensitivity and 94.7% specificity, with kappa = 0.96 (almost perfect agreement). Two samples (one tomato and one cucumber) were marginally positive for TYLCV, while a single smooth luffa sample exhibited a weakly positive reaction for ToLCNDV via RPA-LFD detection, yet was negative via PCR. The Sanger sequencing of the TYLCV and ToLCNDV PCR amplicons from the four double-positive tomato samples yielded sequences with 99–100% identity to the corresponding TYLCV and ToLCNDV reference sequences (GenBank accession numbers: HQ702862.1 and PV083591.1, Supplementary Table S1), independently confirming the co-infections.

**Table 3:** Detection results of field-collected samples by duplex RPA-LFD protocol in comparison with conventional PCR.

Sample type	Total No.	Duplex RPA-LFD results (no.)				Conventional PCR results (no.)				Agreement rate (%)
		TYLCV	ToLCNDV	Double positive	Negative^*^	TYLCV	ToLCNDV	Double positive	Negative^*^	
Tomato	21	10	5	4	2	9	5	4	3	*95.2%*
Cucurbits	36	2	30	0	4	1	29	0	6	*94.4%*
Total	**57**	**12**	**35**	**4**	**6**	**10**	**34**	**4**	**9**	***94.7%***

^*^Negative: This refers to the absence of detectable TYLCV and/or ToLCNDV. Bold values in the ‘Total' row indicate the overall detection results obtained for all 57 field-collected samples.

**Table 4:** Diagnostic performance of the duplex RPA-LFD assay using conventional PCR as the reference method.

Statistic	TYLCV	ToLCNDV
True positive (RPA+/PCR+) (TP) ^a^	14	38
False positive (RPA+/PCR–) (FP) ^a^	2	1
False negative (RPA–/PCR+) (FN) ^a^	0	0
True negative (RPA–/PCR–) (TN) ^a^	41	18
Sensitivity ^b^ (%)	100	100
Specificity ^b^ (%)	95.3	94.7
Positive predictive value (PPV) ^b^ (%)	87.5	97.4
Negative predictive value (NPV) ^b^ (%)	100	100
Cohen's kappa ^c^	**0.91**	**0.96**
Agreement ^c^	almost perfect	almost perfect

^a^Number of samples assigned to each diagnostic category from the 57 field-collected samples. In the Statistic column, the symbols + and – denote positive and negative results of each assay, respectively (e.g., RPA+/PCR+ indicates a sample positive by both the duplex RPA-LFD and conventional PCR). ^b^Sensitivity = TP/(TP+FN) × 100%; Specificity = TN/(TN+FP) × 100%; PPV = TP/(TP+FP) × 100%; NPV = TN/(TN+FN) × 100%. ^c.^Calculated following Cohen (1960) and interpreted according to Landis and Koch (1977) (0.81–1.00 = almost perfect agreement). Double-positive samples (*n* = 4) were counted as positive for both TYLCV and ToLCNDV. Bold values indicate Cohen's kappa, the primary statistic used to assess agreement between the two assays.

## 4 Discussion

Agricultural globalization has led to the trans-regional and cross-continental spread of plant viruses, resulting in endemic or pandemic viral diseases worldwide. Mixed viral infections pose an unpredictable challenge to plant protection and quarantine (Tatineni and Hein, 2023). The present study focused on two notorious begomoviruses, TYLCV and ToLCNDV, which threaten global production of solanaceous (e.g., tomato) and cucurbit (e.g., cucumber and melon) crops. Through the use of infectious clones as standardized viral inocula, we developed a duplex RPA-LFD assay as a rapid, sensitive, and user-friendly diagnostic tool for the screening of TYLCV and ToLCNDV, singly or in combination. The construction of an infectious clone of a ToLCNDV strain isolated from China also provides a fundamental inoculation tool to study viral pathogenicity, screen for virus-resistant germplasms, and elucidate virus-host interactions. Although the ToLCNDV Asian clade isolate used in this study exhibited only limited infectivity in the tested tomato cultivars, it successfully co-infected *N. benthamiana* together with the TYLCV infectious clone, thereby fulfilling its primary purpose as a validated positive control for duplex RPA-LFD development.

Our inoculation trials showed that the ToLCNDV clone could effectively infect various cucurbit crops and *N. benthamiana*, causing symptoms similar to those observed in naturally infected cucurbit crops in the field (Mei et al., 2023), but induced only a low-rate and completely asymptomatic infection in tomato. This pattern—systemic infection without visible symptoms in tomato—has also been reported for other cucurbit-derived ToLCNDV isolates of the Asian clade from China, including isolates from Hebei and Jiangsu provinces (Chen et al., 2026; Qiao et al., 2026), suggesting that cucurbit-adapted ToLCNDV strains may exhibit reduced virulence in their original solanaceous host. Consistent with this, European strains (e.g., ToLCNDV-ES) are also known to be poorly infective on tomatoes (Fortes et al., 2016, 2025). The underlying mechanism of this differential host adaptation remains to be elucidated and warrants further investigation. Notably, despite the low infection rate of the cucurbit-derived ToLCNDV infectious clone in tomato under laboratory conditions, TYLCV–ToLCNDV co-infected tomato samples were readily collected from the field during this study. This apparent discrepancy may reflect several factors. First, field ToLCNDV populations in tomato may represent tomato-adapted variants that differ from cucurbit isolates in host preference, as previously documented for begomoviruses (Moriones et al., 2017). Second, the nature of the interaction between TYLCV and ToLCNDV in co-infected tomato—whether synergistic, antagonistic, or neutral—remains unclear and may influence their relative accumulation in host plants (Vo et al., 2022; Fortes et al., 2025). Third, whitefly-mediated transmission under natural field conditions may contribute to higher inoculation efficiency than agroinfiltration (Ruiz et al., 2017). These possibilities, while beyond the scope of the present study, highlight the importance of further work comparing the infectivity of cucurbit- and tomato-derived ToLCNDV isolates. Furthermore, the successful coinfection of *N. benthamiana* with the two infectious clones demonstrates their utility as tools for future studies (Fortes et al., 2025).

The visual-assisted duplex RPA-LFD detection protocol developed here integrates the isothermal and rapid features of RPA with the simplicity and instrument-free advantages of LFD to achieve simultaneous detection of TYLCV, ToLCNDV, or both viruses within 20 min at 39 °C. The employment of two distinct hapten labels (i.e., biotin and DIG) on probes in conjunction with a commercially available dual-test-line LFD strip enables a successful differentiation of the two viruses via independent test lines on the same strip (Jauset-Rubio et al., 2016; Xie et al., 2025). This approach enables the test strip to identify and visualize whether viral suspect samples are infected with one or multiple serious viruses, with which an effective and practical strategy can be implemented to curb the disease progress (Sun et al., 2019). Compared to the detection methods for either TYLCV or ToLCNDV, the duplex RPA-LFD assay significantly improves the diagnostic efficiency with a simple isothermal heating device (e.g., a water bath or heat block set at 39 °C), which is more economical than other thermal cycler-based and electrophoresis-dependent diagnostics (Zhou et al., 2022). In addition, the high specificity ensures the accuracy and sensitivity to meet the needs for early field diagnosis and monitoring (Tamer et al., 2026). Field testing further confirmed that the assay reliably detected TYLCV and/or ToLCNDV in both tomato and cucurbit samples (Table 3), supporting its broad applicability across host types for single- and mixed-infection surveillance.

We have reported a heat-labile uracil-DNA glycosylase-based LAMP-LFD assay for rapid detection of ToLCNDV alone (Qiao et al., 2026). Compared with that singleplex LAMP-LFD, the duplex RPA-LFD developed here offers several practical advantages for diagnosing co-infected viral diseases. Most importantly, it detects TYLCV and/or ToLCNDV simultaneously in a single reaction rather than the ToLCNDV alone. In addition, it operates at a lower temperature (39 °C vs. 60–65 °C for LAMP) and in a shorter time (20 min vs. typically 30–60 min), which reduces energy consumption and better suits field deployment. Furthermore, the RPA design requires only two primers and one probe per target, which is considerably simpler and easier to multiplex than the four-to-six primers required for LAMP. The two assays are thus complementary rather than substitutive: the LAMP-LFD is well suited to highly sensitive single-target ToLCNDV screening, whereas the duplex RPA-LFD is better suited to the rapid simultaneous discrimination of TYLCV–ToLCNDV co-infections. In terms of sensitivity, the reported LODs in this study are analytical LODs determined with purified plasmid DNA and may not directly reflect the actual detection sensitivity on field-infected plant samples, owing to plant genomic background, viral titer, and extraction efficiency. Although the assay was validated using co-inoculated *N. benthamiana* and field samples, the LOD in the background of serially diluted infected-plant DNA remains to be determined.

To further improve the assay's diagnostic robustness, testing on additional samples from more diverse host plants and geographical regions within China is warranted (Rubio et al., 2020; Geiser et al., 2023). In terms of the infectious clone, the primary goal of its biological characterization was to generate defined viral inocula for method development, rather than to provide a comprehensive determination of host range. The observed 14.3% infection rate in inoculated tomato was derived from a limited sample size (*n* = 21) and should therefore be interpreted with caution. Inoculation of ToLCNDV DNA-A or DNA-B separately in *N. benthamiana* or other host plants was not performed since our priority was to construct a functional bipartite clone for diagnostic purposes. Future studies should address these questions through larger-scale and geographically broader field surveillance combined with targeted biological experiments. Systematic monitoring across tomato- and cucurbit-growing regions would help establish more robust estimates of the prevalence and epidemiological significance of mixed infections involving ToLCNDV, whereas mechanistic studies on TYLCV–ToLCNDV interactions would further clarify the biology and ecological implications of ToLCNDV in co-infected hosts.

It should be noted that, while RPA tolerates a limited number of primer-template mismatches (Daher et al., 2015; Lobato and O'Sullivan, 2018), the RPA primers and probes used in this study were designed from conserved regions identified through multiple sequence alignment of 16 TYLCV and 14 ToLCNDV complete genome sequences of Chinese isolates available from GenBank (Supplementary Table S1). This design strategy minimizes the risk of false negatives caused by sequence polymorphisms in field populations. Moreover, although being developed here for detection of two DNA viruses (TYLCV and ToLCNDV) only, the diagnostic method could be further extended to detect other economically important begomoviruses. For RNA viruses, a reverse transcription step (RT-RPA) should be incorporated; adding the RT module into the multiplex detection protocol for simultaneous detection of both DNA and RNA viruses should be feasible and valuable (Mota et al., 2022; Wu et al., 2024). Furthermore, the use of QuickExtract for rapid DNA preparation further simplified the workflow by eliminating the need for centrifugation and reducing the extraction time to approximately 8 min, consistent with the broader trend toward simplified nucleic acid release methods in molecular diagnostics (Alexander, 2016; Zhao et al., 2023). Finally, a smartphone-based imaging application and data analysis can be implemented to digitize and network the testing results (Zhao et al., 2023; Jain et al., 2025). Addressing these limitations and pursuing the suggested future directions will further strengthen the applicability and robustness of the duplex RPA-LFD assay for field diagnostics and epidemiological surveillance.

## Data Availability

The original contributions presented in the study are included in the article/Supplementary material, further inquiries can be directed to the corresponding authors.

## Supplementary material

Supplementary Figure 1 Negative control (non-infected) plants corresponding to the host species shown in Figure 3. Healthy, mock-inoculated plants of *N. benthamiana* (**A**), cucumber (**B**), pumpkin (**C**), zucchini (**D**), watermelon (**E**), smooth luffa (**F**), melon (**G**), and tomato (**H**) showing no viral symptoms.

Supplementary Table 1 Information of ToLCNDV DNA-A and TYLCV isolates used for sequence alignment.
